# Clinical MRI to predict motor and non-motor effects of deep brain stimulation in Parkinson disease

**DOI:** 10.1007/s11547-025-02025-8

**Published:** 2025-06-09

**Authors:** Corrado Campisi, Giovanni Giulietti, Carlo Alberto Artusi, Federico D’Agata, Giovanni Morana, Claudia Ledda, Elisa Montanaro, Mario Coriasco, Leonardo Lopiano, Marco Bozzali

**Affiliations:** 1 ‘Rita Levi Montalcini’ Department of Neuroscience, University of Torino, Via Cherasco 15, 10126 Turin, Italy; 2 Neuroimaging Laboratory, IRCCS Santa Lucia Foundation, Rome, Italy; 3 SAIMLAL Department, Sapienza University of Rome, Rome, Italy; 4 SC Neurologia 2U, AOU City of Health and Science, Turin, Italy

**Keywords:** Parkinson’s disease, Electrical stimulation, MRI, Cognition, Behavior

## Abstract

**Purpose:**

Subthalamic deep brain stimulation (STN-DBS) is a well-established intervention for advanced Parkinson’s disease (PD). Routine neuroimaging can be used to estimate location and volume of activated tissue (VTA), by modeling the type of stimulator and stimulation parameters. We aimed here at developing a strategy based on clinical brain MRI scans to predict motor and non-motor outcomes of STN-DBS.

**Materials and methods:**

We included 25 consecutive patients with advanced PD eligible for STN-DBS. At baseline, patients underwent a comprehensive motor and cognitive/behavioral assessment, and conventional MRI. They underwent STN-DBS surgery, followed by a CT scan. Patients were reassessed 1 year later, while STN-DBS was active. Their neuroimaging data were used to calculate individual VTAs. The voxel-lesion-symptom-mapping (VLSM) toolbox, which allows to associate clinical variables with brain features of interest, was used to investigate associations between changes (in either direction) of motor, cognitive/behavioral scores between baseline and follow-up, and VTA subregions. Six newly enrolled patients were used to test the predictive value of this approach at a single subject level.

**Results:**

VLSM analysis (*p* values corrected for multiple comparisons < 0.05) identified specific VTA subclusters associated with improved bradykinesia, verbal fluency, and mood state, and some others associated with worsening of tremor, long-term memory, and apathy. When considering cognitive/behavioral changes, an effect of hemisphere lateralization was observed, with modulation of the right basal ganglia being associated with symptoms’ worsening, and left-side modulation associated with improvements. VTA subclusters predictive for clinical changes were mostly located outside the STN, indicating the importance of networks over single nuclei simulation.

**Conclusion:**

This approach suggests a possible way to personalize surgical planning, DBS-implant choice, and stimulation programing in the framework of precision medicine.

## Introduction

Bilateral Subthalamic–Deep-Brain Stimulation (STN-DBS) is a well-established device-aided intervention for the treatment of complicated Parkinson's disease (PD) [1]. STN-DBS has proven efficacious in ameliorating fluctuations of levodopa-responsive symptoms alongside patients’ quality of life [2]. Nonetheless, STN-DBS outcome remains hardly predictable before surgery due to a huge individual variability despite application of strict selection criteria [3]. Lead implantation is preferentially conducted in awake surgery based on anatomical guidelines and neurophysiological recordings. After surgery, stimulation parameters are set up and adjusted in combination with Levodopa Equivalent Daily Dose (LEDD). Main clinical aspects that are used to define the outcome of STN-DBS as unsatisfactory include the persistence of motor symptoms and fluctuations, and/or appearance of stimulation-related side effects. However, assessing patients’ STN-DBS eligibility by merely controlling for age, motor symptom response to levodopa therapy, the presence of axial symptoms, and the absence of cognitive impairment [3], is likely insufficient for a high confidence prognosis of STN-DBS outcome [4]. Importantly, STN-DBS modulates also cognition, mood state and sleep regulation [5]. A better understanding of STN-DBS effects on specific brain structures would strongly inform surgical planning, selection of electrode type (i.e., standard vs. directional), and stimulation parameters according to each individual clinical features, in the framework of personalized medicine. Nowadays, there are available tools to define on individual MRI scans the brain regions that are stimulated by STN-DBS implants (i.e., volume of tissue activated; VTA), based on algorithms that model electrodes’ type and stimulation parameters [6].

Image analysis approaches can be used to model STN-DBS stimulation voxel-wise [7–11], thus predicting the effects of specific electrode types and locations. The general idea relies on bringing images from patients into a common space, mapping their VTAs and classifying the VTA voxels based on their association with clinical improvement. Earlier applications of such an approach based on a variety of different methodologies focused on patients with PD and/or dystonia [7–12].

In the current study, we used an approach similar to that proposed by Reich et al. [11] to identify, in a group of consecutive patients with PD who underwent STN-DBS, anatomical subregions of their VTAs that might account for positive or negative motor and non-motor clinical outcomes. Importantly, for the first time we extended the investigation beyond motor outcomes, to include neuropsychological and behavioral measures. Based on the patients’ presurgical MRI and post-surgical CT scans, we estimated individual VTAs and tested, using a voxel-wise approach, the probability of each VTA subregion to be associated with measures of STN-DBS efficacy or inefficacy at 1-year interval. As a proof of concept, we also tested this model ability to predict individual STN-DBS outcomes in a small sample of six newly enrolled patients, demonstrating a good agreement between predictions and clinical outcome.

## Materials and methods

### Study design and population

Fifty-five patients with advanced PD were consecutively screened for eligibility to bilateral STN-DBS at the Movement Disorder Clinic of “Città della Salute e della Scienza” University Hospital (Turin, Italy). As detailed below, 25 of them [M/F: 21/4; mean (SD) age: 62.3 (8.0) years; mean (SD) disease duration: 11.7 (4.5) years] resulted eligible for STN-DBS according to current clinical criteria [3]. Their mean (SD) Hoen & Yahr score at baseline was 2.3 (0.6) on no medication (Med-OFF), and 1.9 (0.5) on medication (Med-ON). As part of the study, in addition to routine procedures (i.e., clinical, neuropsychological and behavioral evaluation; presurgical MRI scanning; post-surgical CT scanning) patients were requested to undergo an extra clinical, neuropsychological and behavioral assessment at 1-year interval. Ethics approval for the study was obtained from the Local Ethics Committee (protocol number: 0068394). Written informed consent from all participants was obtained soon after their clinical selection for STN-DBS, before surgery.

All patients underwent bilateral STN-DBS implantation according to a well-established procedure [13]. Immediately after surgery, they underwent a brain CT scan to rule out surgical complications. One to two weeks after surgery, bilateral STN-DBS parameters of stimulation were set up individually to obtain the best motor response.

#### Baseline data collection

Presurgical motor evaluation was performed in each patient through a levodopa challenge test, which was always carried out in the morning. Patients were evaluated in Med-OFF (12 h after their last levodopa administration), and in Med-ON condition (40 min after administration of a levodopa challenge dose, consisting of 1.5 × their usual dose). The MDS-UPDRS [14] part III was used to assess the motor response to levodopa, while motor complications were evaluated using a subset of items from MDS-UPDRS part IV. In addition to the global MDS-UPDRS part III score, a set of sub-scores (details are reported in Table’s 1 legend) were considered to estimate changes in bradykinesia, rigidity, tremor, axial symptoms. Neuropsychological and behavioral assessments were performed in Med-ON condition, in morning time. In addition to the Mini Mental State Examination (MMSE) score as measure of global cognition, an extensive neuropsychological battery was employed to explore all major domains: (1) *Verbal Long-term Memory*: Story Recall Test, Paired associated Learning Test, and Rey Auditory Verbal Learning Test; (2) *Short-term and Working Memory*: Digit Span; Disyllabic Word Repetition Test, and Corsi’s Block Tapping Task (forward); (3) *Non-verbal reasoning*: Raven’s Colored Progressive Matrices; (4) *Attention and Executive Functions*: Digit Cancelation Test, Trail Making Test A and B, Phonemic and Semantic Verbal Fluency Tests, Frontal Assessment Battery, and Nelson Modified Card Sorting Test; (5) *Visuo-spatial abilities*: Benton Judgment of Line Orientation Test. References for these cognitive tests can be found elsewhere [15, 16]. For the behavioral assessment, patients were administered the Beck Depression Inventory (BDI), the State-Trait Anxiety Inventory X Form (STAI X) [17], the Marin’s Apathy scale [18], the Parkinson’s Disease Questionnaire (PDQ-39) [19], the questionnaire for impulsive compulsive behaviors in PD (QUIP) [20], the Barratt’s Impulsiveness Scale (BIS) [21], the Parkinson’s Disease Sleep Scale 2 (PDSS 2) [22], the Epworth Sleepiness Scale (ESS) [23], and the REM sleep behavior disorder questionnaire (RBDSQ) [24].

**Table 1:** Neuropsychological, behavioral and motor assessment obtained at baseline and follow-up

Neuropsychological domain scale			Baseline Med-ON	Follow-up Stim-ON/Med-ON	*p* value*	Cohen’s D
*Global cognition*						
Mini Mental State Examination			29.0 (0.8)	–	–	–
*Verbal Long-term Memory*						
Story Recall Test			16.7 (4.7)	16.8 (4.2)	0.47	0.019
Paired associated Learning Test			12.7 (2.8)	13.2 (2.9)	0.06	-0.322
Rey Auditory Verbal Learning Test					**0.05**	− 0.329
Immediate recall			43.3 (8.6)	46.3 (10.2)		
Delayed recall			9.3 (2.4)	10.0 (2.5)	**0.05**	− 0.327
*Short-term and Working Memory*						
Digit Span (forward)			4.2 (0.8)	3.7 (0.8)	**0.005**	0.155
Disyllabic Word Repetition Test			4.2 (0.8)	3.7 (0.8)	**0.005**	0.565
Corsi’s Block Tapping Task (forward)			4.8 (0.9)	4.6 (0.9)	0.20	0.168
*Non-verbal reasoning*						
Raven’ Colored Progressive Matrices			31.9 (2.2)	31.4 (3.9)	0.26	0.133
*Attention and Executive Functions*						
Digit Cancelation Test			46.9 (5.8)	46.0 (5.1)	0.24	0.146
Trail Making Test A			22.6 (7.3)	28.3 (13.8)	**0.02**	− 0.42
Trail Making Test B			63.7 (48.7)	94.8 (78.9)	**0.05**	− 0.511
Trail Making Test B–A			40.2 (46.4)	67.3 (76.6)	**0.04**	− 0.456
Phonemic Verbal Fluency			43.1 (14.3)	37.8 (12.4)	**0.005**	0.564
Semantic Verbal Fluency			23.5 (6.0)	21.4 (5.3)	**0.02**	0.427
Frontal Assessment Battery			16.1 (1.5)	16.0 (2.0)	0.42	0.04
Nelson Modified Card Sorting Test			1.7 (2.8)	1.7 (1.9)	0.95	0.028
Visuo-spatial Abilities						
Benton Judgment of Line Orientation Test			25.8 (3.9)	24.6 (4.7)	0.10	0.374
*Behavioral Domains*						
Beck Depression Inventory			9.6 (5.5)	8.0 (4.8)	**0.03**	0.413
State-Trait Anxiety Inventory (STAI)						
STAI X1			39.5 (8.2)	41.2 (9.0)	0.49	0.005
STAI X2			42.1 (8.2)	41.1 (9.7)	0.18	0.2
Marin’s Apathy scale			9.5 (5.3)	13.7 (6.5)	**0.009**	− 0.547
The Parkinson disease Questionnaire			26.4 (16.0)	21.5 (12.9)	**0.05**	0.357
Questionnaire for impulsive compulsive behaviors in PD (QUIP)			16.6 (13.9)	16.0 (14.7)	0.42	− 0.044
Barratt’s Impulsiveness Scale			59.7 (7.7)	61.5 (7.7)	0.104	− 0.319
Parkinson’s Disease Sleep Scale 2			19.8 (10.4)	14.8 (9.6)	**0.02**	0.465
Epworth Sleepiness Scale			8.5 (3.9)	6.7 (3.5)	**0.005**	0.659
The REM sleep behavior disorder screening questionnaire			4.3 (1.9)	3.9 (2.5)	0.123	0.267
	Side	Baseline		Follow-up	*p* value*	Cohen’s D
		Med-OFF		Stim-ON/Med-OFF		
*MDS-UPDRS III*						
Total score	–	47.7 (11.8)		32.3 (12.5)	** < 0.001**	1.383
Bradykinesia^(1)^	B	22.2 (5.7)		16.5 (7.0)	**0.001**	1.118
	R	10.4 (3.0)		7.8 (3.4)	**0.002**	1.07
	L	11.0 (3.4)		8.2 (3.9)	**0.005**	0.987
Rigidity^(2)^	B	8.9 (3.5)		6.1 (3.5)	** < 0.001**	1.129
	R	3.4 (1.5)		2.4 (1.7)	**0.009**	0.917
	L	3.6 (1.8)		2.5 (1.6)	**0.007**	0.941
Tremor^(3)^	B	6.7 (5.9)		3.2 (4.0)	**0.007**	0.938
	R	2.0 (1.9)		0.9 (1.3)	**0.015**	0.868
	L	2.6 (2.9)		1.3 (1.6)	**0.03**	0.798
*MDS-UPDRS II/III*						
Axial symptoms^(4)^	–	8.6 (2.9)		5.7 (2.8)	** < 0.001**	1.395
*MDS-UPDRS IV*					** < 0.001**	
Dyskinesias^(5)^	–	1.8 (1.2)		0.8 (0.9)	**0.001**	1.247
Off-state^(6)^	–	1.5 (0.9)		0.8 (0.7)	** < 0.001**	1.135
*INTERVENTION*						
STN-DBS						
CI (mA)	R	–		2.58 (0.6)	–	–
	L	–		2.62 (0.6)	–	–
LEDD (mg)	–	1145.4 (268.9)		585.8 (230.8)	** < 0.001**	1.952

^*^Paired *t* test

*Abbreviations CI* current intensity, *MDS-UPDRS III* MDS-Unified Parkinson's Disease Rating Scale, *LEDD* Levodopa Equivalent Daily Dose

^1^Bradykinesia: evaluated by summing MDS-UPDRS part III sub-scores on items 3.4 a–b (i.e., right and left finger tapping), 3.5 a–b (i.e., right and left hand movement), 3.6 a–b (i.e., right and left hand pronation), 3.7 a–b (i.e., right and left toe tapping), 3.8 a–b (i.e., right and left leg agility), and 3.9 (i.e., arising from chair)

^2^Rigidity: evaluated by summing MDS-UPDRS part III sub-scores on items 3.3 a–e (i.e., neck, right and left upper and lower extremities)

^3^Tremor: evaluated by summing MDS-UPDRS part III sub-scores on items 3.15 a–b (i.e., right and left hand postural tremor), 3.16 a–b (i.e., right and left hand kinetic tremor), 3.17 a–d (i.e., right and left, upper and lower limb rest tremor amplitude) and 3.18 (i.e., constancy of resting tremor)

^4^Axial symptoms evaluated by summing MDS-UPDRS part II and III sub-scores on items 2.13 (i.e., freezing), 3.1 (i.e., speech), 3.3 (i.e., neck rigidity), 3.9 (i.e., arising from chair), 3.10 (i.e., gait), 3.12 (i.e., postural stability), 3.13 (i.e., posture)

^5^Time spent with Dyskinesias (MDS-UPDRS part IV; sub-scores 4.1)

^6^Time in OFF (MDS-UPDRS part IV; sub-score 4.3)

Motor assessments were first performed at baseline (before surgery) in med-OFF (i.e., at least 12 h after the last levodopa administration), while neuropsychological and behavioral ones were performed at baseline in Med-ON condition. At 1-year follow-up, the same motor assessments were repeated in med-OFF, but with active bilateral STN-DBS (Stim-ON) while neuropsychological and behavioral ones were repeated in Med-ON condition and with active bilateral STN-DBS (Stim-ON)

All scores reported in the table are expressed in mean (SD)

Appropriate references are quoted in the Methods section

Statistically significant differences are highlighted in bold characters (*p* equal or inferior to 0.05)

Within 2 weeks after motor, neuropsychological and behavioral assessments, all patients underwent presurgical MRI scanning at 3 T (Philips Ingenia), including the following three-dimensional acquisitions: (1) T1-weighted scan (TR = 7.9 ms; TE = 3.5 ms; Matrix = 240 × 240 × 190, in–plane FOV = 240 × 240 mm^2^, slice thickness = 1.0 mm; ETL = 213); (2) T2-weigted scan (TR = 3000 ms, TE = 198 ms, Matrix = 240 × 240 × 190, in–plane FOV = 240 × 240 mm^2^, slice thickness = 1.0 mm, ETL = 104); fluid-attenuated-inversion-recovery (FLAIR) scan (TR = 4.800 ms, TE = 340 ms, TI = 1650 ms, same matrix and FOV as in T2-weighted scan, slice thickness = 1.0 mm, ETL = 199).

Post-surgical head CTs were performed helically on a Siemens multidetector CT scanner (Matrix = 512 × 512 × 189, slice thickness = 1.0 mm).

#### Follow-up data collection

One year after STN-DBS surgical implantation, all patients were requested to attend a dedicated session to repeat all assessments performed at baseline, with the exception of MRI.

For the motor assessment, evaluation of all MDS-UPDRS part II/III and IV scores and sub-scores was repeated in Med-OFF, Med-ON condition, and in stimulation-on (Stim-ON) condition.

Neuropsychological and behavioral assessment were also repeated, this time in Med-ON and Stim-ON condition.

For each subject, the ongoing STN-DBS stimulation parameters were recorded and used for VTA estimation on individual presurgical MRI data.

#### Neuroimaging data analysis

**Fig. 1:**
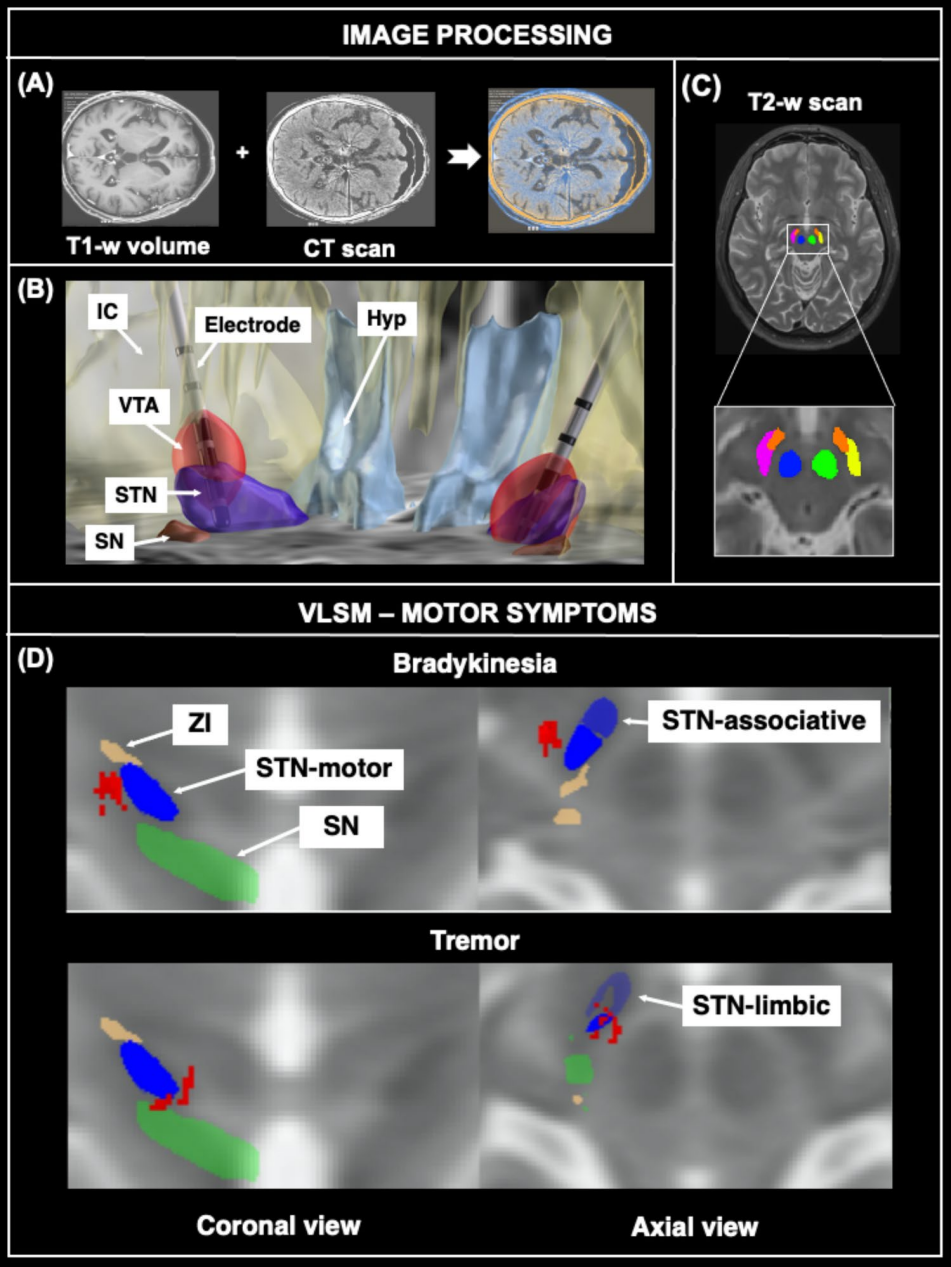
Image processing and imaging associations with motor symptom changes. Principal steps of image processing applied to a single case and VSLM results for association between changes in motor symptoms (between baseline and follow-up) and voxels of DBS activation. Panel A shows the coregistration step between a T1-weighted volume collected at baseline and the correspondent CT scan obtained immediately after surgery. Panel B shows electrode localization based on the hyperdensity detectable on the CT scan, after correction for brainshift and image normalization. Panel C shows modeling of stimulation parameters (i.e., current intensity) to obtain estimation of each VTA, which is overlayed on the basal ganglia map obtained by automatic segmentation of each individual T2-weighted scan. Panel D shows VTA subregions (red areas) whose activation was with improvement of bradykinesia and worsening in tremor. Nuclei in the picture were obtained by averaging those resulting from segmentation of T2-weigthed images from all studied subjects. Color legend: Red = clusters of activation; blue = subthalamic nucleus, divided in its motor (dark blue), associative (intermediate blue) and limbic portion (pale blue); green = substantia nigra; light brown = zona incerta. *Abbreviations* Hyp = Hypothalamus; IC = internal capsule; SN = substantia nigra; STN = subthalamic nucleus; VTA = volume of tissue activated; ZI = zona incerta

Neuroimaging data were first processed using the MATLAB® toolbox Lead-DBS v2.6 (www.lead-dbs.org). For each subject, T1-weighted images were coregistered to post-surgical head CT using ANTs (stnava.github.io/ANTs/), and to T2-weighted and FLAIR images using SPM12 (www.fil.ion.ucl.ac.uk/spm/software/spm12/). Normalization to MNI_ICBM_2009b_NLIN_ASYM (www.bic.mni.mcgill.ca/ServicesAtlases/ICBM152NLin2009/) space was carried out using ANTs, which was followed by nonlinear deformation of the region of interest based on the subcortical definition mask computed as defined in Schönecker et al. [25] to account for brainshift. Electrodes’ position was determined on the post-surgical CT images and reconstructed using PaCER automatic algorithm. Each individual VTA was obtained by entering the ongoing stimulation parameters (average values are reported in Table 1). To visualize each VTA, we used Fastfield [26] as estimation algorithm, setting the E-field threshold to 0.19 V/mm and brain conductivity to 0.1 S/mm. An example of this processing is shown in Fig. 1.

Using the online-tool volBrain-pBrain (volbrain.upv.es), T2-weighted images from all subjects were segmented to obtain the principal subcortical gray matter nuclei: the STN, the substantia nigra (SN), and the red nucleus (RN). Average maps of these nuclei were used to overlap results from the voxel-based lesion-symptom mapping (VLSM) analysis (see below). For a better characterization of nuclei subfields, the DISTAL atlas [27] was employed after checking for satisfactory matching with nuclei obtained by segmentation of our patients’ T2-weighted scans.

#### Voxel-based lesion–symptom mapping

VLSM is a toolbox operated in MATLAB that carries out voxel-wise statistical analyses of imaging data. VLSM was originally developed to test for associations between the anatomical distribution of brain lesions and continuous clinical variables (e.g., symptom severity or performance on specific clinical/neuropsychological tests) [28]. We employed here the statistical algorithm of VLSM to associate clusters of stimulation (instead of brain lesions for which the software was originally developed) within our patients’ VTAs and measures of clinical outcome (i.e., changes between baseline and follow-up). Then, given a clinical variable of interest (e.g., bradykinesia, rigidity, etc.), we performed, using VLSM analysis, a series of two-sample *t* tests, one for each voxel included in the VTAs from all patients. The groups were defined based on whether the given voxel belonged or not to each patient VTA (i.e., patients whose VTA included the voxel were assigned to group A [stimulated], while patients whose VTA did not, were assigned to group B [non-stimulated]). Generally, the two groups were different for each *t* test. The *t* tests in which either group included less than four patients, were discarded in order to maintain a reasonable level of statistical power. All motor and non-motor variables obtained from neuropsychological, behavioral and motor assessment (see Table 1) whose scores showed a statistically significant modification (in either direction) between baseline and 1-year follow-up, were converted into Z scores, and their algebraical difference was used for VLSM analyses. We therefore used each Z score difference to quantify an improvement or worsening from baseline. The resulting statistical maps were cluster-level corrected for multiple comparisons using permutation tests with 1000 repetitions, accepting *p* values of less than 0.05 as statistically significant. Permutation tests are based on a nonparametric resampling approach that provides elegant solutions for numerous statistical issues. Statistics is compared to a null distribution derived from the dataset of interest rather than from a parametric distribution. In VLSM analyses, patient age, sex, and disease duration were always entered as covariates of no interest. Additionally, when exploring neuropsychological and behavioral effects of DBS, LEDD reductions and MDS-UPDRS part III changes were entered as covariates of no interest. This choice was made to account for the levodopa effect on neuropsychological and behavioral symptoms, considering the impact of medication on patient motivation and motor performance.

To improve the statistical power of VLSM analysis of motor symptoms, images of those subjects who exhibited the right part of the body as the most affected were flipped from the left to the right hemisphere, similarly to a recent study [29]. This means that in all patients, the most affected side of the body was artificially considered as the left side. This procedure was not applied to VLSM analyses of cognitive/behavioral symptoms considering the importance of hemispheric specialization in higher level functions.

#### Model predictability

We tested our model on six new consecutive patients who underwent STN-DBS implantation and received their first follow-up at 6 months. Using Lead-DBS [6], we first defined every patient’s VTAs and calculated the distance between their own VTA center and each cluster’s center resulted from VLSM analyses. Then, for each set of distances between patients’ VTAs and VLSM clusters we calculated the median value. We used these medians as cutoff values to define two groups for each analyzed domain: a closer and a farther stimulated group. Then, we calculated the percentage change between each patient baseline and follow-up clinical and neuropsychological/behavioral scores. Finally, we compared mean changes between the two groups to assess whether the new patients were following the model we developed, considering the closer group to be following the improvement or worsening in scores of the analyzed cluster. On this basis, we defined, for each clinical change, the predictive value of VLSM.

## Results

All patients reported motor improvement after STN-DBS in the absence of surgical adverse events (Table 1). In both timepoints (baseline and follow-up), motor assessments were performed in Med-OFF condition. This means that within the limitations of a clinical study (i.e., the absence of electrodes in place at baseline), all UDPRS part III changes reflect with good approximation the pure effect of STN-DBS. Importantly, patient time spent with Dyskinesias and off-state (UPDRS IV sub-scores) was significantly reduced at follow-up, consistent with a significant reduction of LEDD.

Cognitive and behavioral effects of STN-DBS were evaluated comparing patients’ performance between baseline and follow-up in Med-ON state, thus exploring the add-on effect of STN-DBS (Table 1). Significant improvements were observed in long-term memory only, while worsening was observed in working memory and executive functions. From a behavioral viewpoint, STN-DBS induced a significant improvement in mood state, impulsivity, sleepiness, and sleep-related disorders. Conversely, worsening in apathy symptoms was observed at follow-up compared to baseline. Consistent with the approach used for VLSM, we also run an additional analysis based on the general linear model to covariate for the contribution of LEDD and MDS-UPDRS III changes on patient performance on cognitive/behavioral tests. No cognitive or behavioral change between baseline and follow-up survived this statistical approach.

Average segmentation of the basal ganglia from our patients’ T2-weighted scans showed a satisfactory matching with those provided in DISTAL atlas [27].

VLSM analysis revealed a significant association between stimulation of a cluster located in the ansa lenticularis and into the motor portion of STN, and patient improvement of bradykinesia. Additionally, stimulation of a cluster located in the SN, the motor portion of STN, and the ansa lenticularis was associated with worsening of tremor. These findings are summarized in Table 2 and illustrated in Fig. 1d.

**Table 2:** Results of VLSM analyses for the motor, cognitive and behavioral domains

Domain	Clinical effect	Hemisphere	Size	Coordinates			*p* value
Symptom/test							
Anatomical localization				*x*	*y*	*z*	
*Motor*							
Bradykinesia							
Lateral AL; Dorsolateral-posterior motor STN	Improvement	–	163	14	− 12	− 9	**0.006**
Tremor							
Antero-dorsal SN; ventromedial motor STN; medial AL	Worsening	–					
			129	8	− 15	− 8	**0.028**
*Cognitive*							
Semantic Verbal Fluency							
Posterior motor STN; dorsal SN; central AL	Improvement	L	236	− 16	− 14	− 9	**0.01**
RAVLT-delayed recall							
Medial associative-STN; dorsal limbic-STN; medial AL	Worsening	R	143	8	− 14	− 10	**0.023**
TMT B-A							
Lateral AL; Central ZI	Worsening	R	108	14	− 10	− 3	**0.026**
Behavioral							
Beck Depression Inventory							
Central ZI; LF; Dorsal motor STN	Improvement	L	288	− 12	− 14	− 3	**0.005**
Marin’s Apathy scale							
Dorsal SN; medial AL; medial motor STN	Worsening	R	106	10	− 15	− 10	**0.05**

*AL* ansa lenticularis; *LF* lenticular fasciculus; *RAVLT* Rey Auditory Verbal Learning Test; *TMT* Trail Making Test; *SN* Substantia Nigra; *STN* subthalamic nucleus; ZI zona incerta

Volume of tissue activated (VTA) clusters whose stimulation resulted associated with changes in motor symptoms or cognitive/behavioral measures between baseline and follow-up

Coordinates refer to the MNI (Montreal Neurological Institute) standard space. Cluster size is expressed in number of voxels

Statistically significant differences are highlighted in bold characters (*p* equal or inferior to 0.05)

**Fig. 2:**
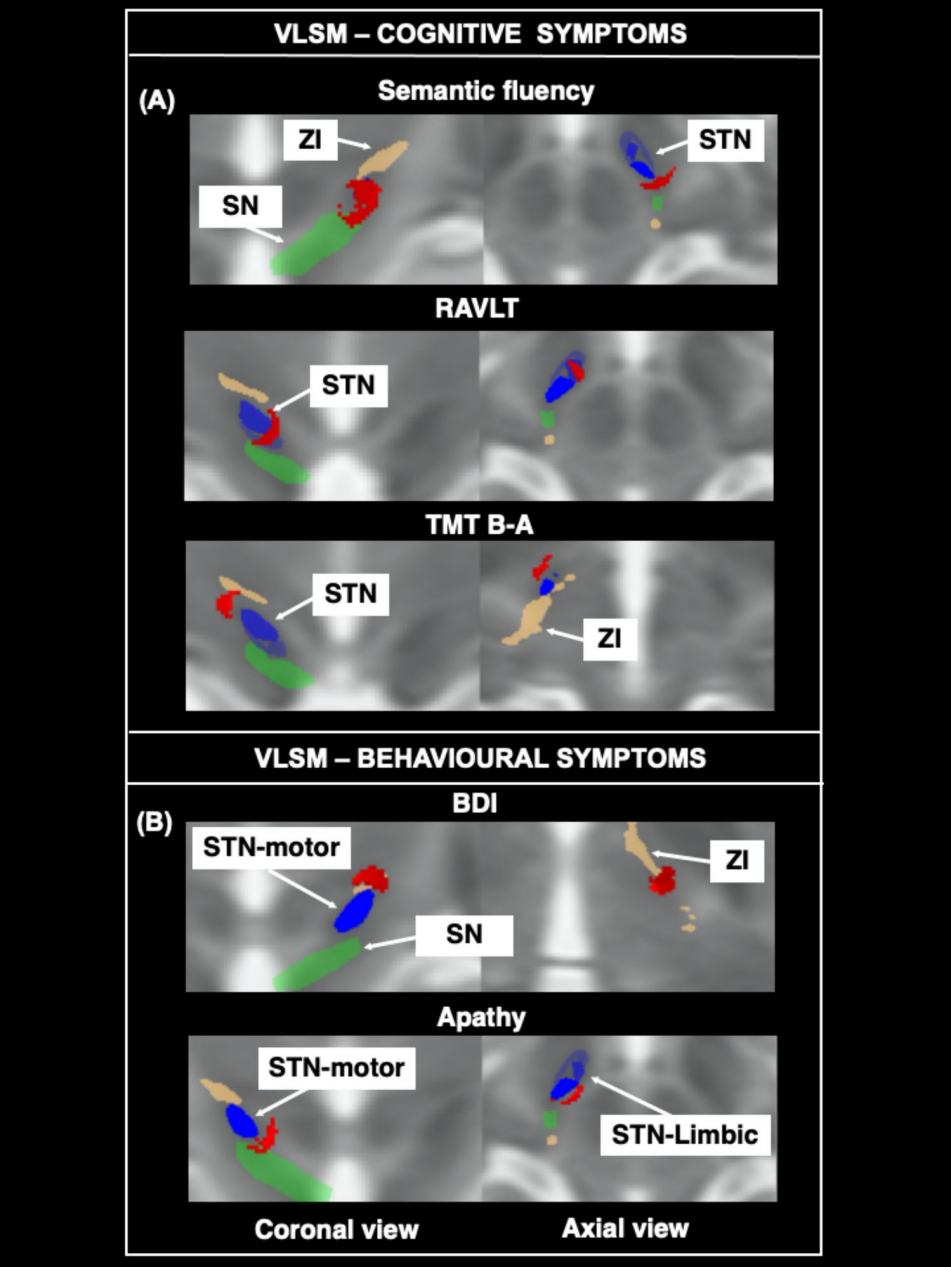
Imaging associations with cognitive and behavioral changes. VLSM results for association between changes in cognitive and behavioral measures (between baseline and follow-up) and voxels of DBS activation (red areas). Panel A shows the clusters associated with improvement of verbal fluency, worsening in episodic memory and worsening in executive functions. Panel B shows the clusters associated with improvement in depression symptoms, and worsening in apathy. Nuclei in the picture were obtained by averaging those resulting from segmentation of T2-weigthed images from all studied subjects. Color legend: Red = clusters of activation; blue = subthalamic nucleus (STN) divided in its motor (dark blue), associative (intermediate blue), and limbic portion (pale blue); green = substantia nigra (SN); light brown = zona incerta (ZI). Abbreviations: BDI = Beck Depression Inventory; RAVLT = Rey Auditory Verbal Learning Test; TMT = Trail Making Test

When considering cognitive measures (Table 2 and Fig. 2a), VLSM showed an association between stimulation of the motor portion of the STN, the SN and ansa lenticularis from the left hemisphere, and improvement in patient scores on semantic fluency. Conversely, patient worsening in long-term memory was associated with stimulation of the right associative and limbic parts of STN, and the right ansa lenticularis. Moreover, worsening in executive functions was found associated with stimulation of the right ansa lenticularis and zona incerta.

When considering behavioral measures (Table 2 and Fig. 2b), improvements in depression were found associated with stimulation of the left zona incerta, the lenticular fasciculus and motor STN. Conversely, worsening in apathy symptoms was observed in association with stimulation of the right substantia nigra, ansa lenticularis and STN motor portion.

The small sample of newly recruited patients (*n* = 6) was used for a proof-of-concept test. Based on the scores’ difference between baseline and follow-up, we obtained two groups dividing the sample according to their median distance from each VSLM significant cluster. For each clinical domain, we report here the percentage of improvement/worsening of the patient subgroup whose VTA center was closer to each VLSM cluster (against those patients whose VTA center was farther). Bradykinesia: 61% vs. 42%, RAVLT 8% vs. 41%, TMT B-A −44% vs −25%, apathy −354% vs −42%, Semantic Verbal Fluency −6.5% vs −7.1%, tremor 27% vs. 0%, BDI −12.5% vs. 20%.

## Discussion

Nowadays, patient selection for STN-DBS is based on strict clinical criteria that are mostly focused on patient response to the levodopa challenge test alongside exclusion of relevant cognitive and behavioral impairments. STN-DBS electrodes are currently implanted according to anatomical and neurophysiological guidelines [30], and stimulation parameters are adjusted based on clinical assessment of patient motor symptoms. The current study collocates in a vast literature bloomed in the last decade, which aimed at investigating the mechanism of DBS [12] through the use of voxel-wise methods of image analyses. VLSM [28] is a toolbox that was previously introduced to investigate associations between patient brain lesions and clinical deficits. Adopting VLSM’s statistical algorithm, we show here a possible strategy based on routinely obtained neuroimaging data, to personalize the type of electrode choice and anatomical location (surgical planning), alongside adjustments of stimulation parameters according to each individual pattern of symptoms. Despite the need for further studies based on larger populations of patients to produce a reliable functional anatomical atlas for VTA, the approach described here indicates VTA subregions whose stimulation associates with a predominant modulation of specific symptoms. Importantly, our findings highlight not only modulation of motor symptoms, but also effects on patient cognition and behavior. These findings suggest that DBS programming should account not only for modulation of STN but also for modulation of surrounding fibers, whose stimulation may affect global clinical outcome. This aspect is becoming increasingly relevant considering the current use of neuroimaging data for identification of the best stimulation contact at individual level. Moreover, continuous technological advances in implantable devices will allow a more flexible manipulation of stimulation parameters for an optimal target tailoring in the framework of personalized medicine.

All patients included in this study showed a substantial improvement of motor symptoms, as shown by their MDS-UPDRS II/III and IV scores. However, when looking at the effect of stimulation in every voxel of their VTAs, specific patterns of symptom improvement or worsening were identified. Overall, our data confirm the complexity of networks that are modulated by subthalamic stimulation and, most importantly, their impact on specific brain functions and quality of life. According to our findings, the best motor improvement (i.e., bradykinesia) is obtained when stimulating an area close to another one which, in contrast, causes worsening of executive functions (i.e., TMT B-A). This means that tailoring the stimulation target not only based on motor functions, should account for a more comprehensive clinical profile of each patient, in the framework of personalized medicine. Moreover, we identified strong associations between cognitive and behavioral symptoms and the stimulated hemisphere. Left hemisphere stimulation led to a general improvement of symptoms, while right hemisphere stimulation led to a general worsening. According to our VLSM findings, stimulation of the posterior dorsolateral part of the STN and the lateral part of the ansa lenticularis, associates with improvement of bradykinesia, while a more ventromedial stimulation of STN (together with stimulation of the medial part of the ansa lenticularis and the anterodorsal part of the substantia nigra) associates with worsening of tremor. The former finding seems particularly robust considering its anatomical similarity to that reported in a previous study [29] in association with improved bradykinesia. The latter finding was not reported before, and requires further validation.

When considering the DBS effect on cognitive and behavioral functions, we observed that voxels’ activation within the left VTA associates with improvement of depression and verbal fluency. Conversely, voxels’ activation within the right VTA associates with worsening of long-term memory, executive functions and apathy. In more detail, we found that stimulation of the left zona incerta (dorsal to STN) associates with improvement of depression. Such an effect may be due to modulation of the left dorsolateral prefrontal cortex, in agreement with a general beneficial effect of stimulation of the zona incerta on higher level dysfunctions [31]. This finding has a potential clinical impact considering the increased risk of young patients to develop post-surgical depression with suicidal attempts [5]. The improvement of semantic fluency when stimulating the left hemisphere is somehow in contrast with previous DBS findings [32]. On the other hand, a detailed comprehension of the frontal contribution to semantic fluency still needs to be fully clarified.

Consistent with our findings, worsening in long-term memory as an effect of DBS was recently shown in a systematic review [33]. Our study helps clarify some neurobiological aspects of this effect, by identifying a cluster of stimulation that is located between the associative and limbic portion of the STN. These STN subfields might indeed be, respectively, implicated in specific long-term memory mechanisms, such as memory storage (associative networks) and retrieval (the limbic system).

We found worsening of patient performance at the Trail Making Test B-A as associated with stimulation of the zona incerta. This finding is consistent with a previous report in PD patients [34], which highlighted an association (at 2-year follow-up) between DBS applied to the zona incerta and decline in Attention and Executive Functions.

Finally, our study revealed an association between worsening in apathy symptoms and stimulation of the right dorsal substantia nigra, medial ansa lenticularis, and the medial part of motor STN. We interpret this effect as due to modulation of the limbic network as previously suggested by others [35].

Within the limitation of a semiquantitative approach applied to a small group of six newly enrolled patients, which does not reach sufficient statistical power to allow generalizability, we observed a good concordance between VLSM predictions on most symptoms and the actual pattern of stimulation in five out of seven considered domains. For generalization of results and definition of test’s sensitivity, specificity and accuracy, a larger sample size is needed.

Main limitations of this study are the relatively small sample size and the single center design, which require future confirmatory studies on larger populations. Nonetheless, we tested here a new simple method of MRI based on clinical datasets to clarify the modulatory effects of DBS on neuronal circuits. Possibly, this approach might have a direct translational potential to improve STN-DBS intervention based on a more comprehensive clinical picture exhibited by individual patients.

## Abbreviations

STN-DBS: Subthalamic nucleus deep brain stimulation
PD: Parkinson’s disease
VTA: Volume of tissue activated
VLSM: Voxel-based lesion-symptom mapping
CAPSIT-PD: Core assessment program for surgical interventional therapies in Parkinson’s disease
LEDD: Levodopa Equivalent Daily Dose
RAVLT: Rey Auditory Verbal Learning Test
TMT: Trail Making Test
SN: Substantia Nigra
RN: Red nucleus
STN: Subthalamic nucleus
MDS-UPDRS III: MDS-Unified Parkinson's Disease Rating Scale
